# Increased quality of life in patients with stroke during the COVID-19 pandemic: a matched-pair study

**DOI:** 10.1038/s41598-021-89746-8

**Published:** 2021-05-13

**Authors:** Li Zhao, Xiaoshi Yang, Fengzhi Yang, Guoyuan Sui, Yi Sui, Bing Xu, Bo Qu

**Affiliations:** 1 School of Public Health, China Medical University, No.77 Puhe Road, Shenyang North New Area, Shenyang, Liaoning Province China; 2 Key Laboratory of Ministry of Education for Traditional Chinese Medicine Viscera-State Theory and Applications, Liaoning University of Traditional Chinese Medicine, Shenyang, China; 3 Department of Neurology, Shenyang First People’s Hospital, Shenyang Medical College, Shenyang, China

**Keywords:** Psychology, Diseases, Medical research, Neurology

## Abstract

Patients with stroke are likely to experience impaired health-related quality of life (QOL), especially during the COVID-19 pandemic. This study aimed to evaluate the QOL of Chinese patients with stroke during the pandemic and explore the associated variables. A matched-pair, multicenter survey was conducted before and during the COVID-19 pandemic. Questionnaires including the 36-item Short-Form Health Survey (SF-36), the Activities of Daily Living (ADL) scale, and the Questionnaire about the Process of Recovery (QPR) were used. A total of 172 matched pairs (344 patients) were recruited in this study. Hierarchical multiple regression analysis was performed to analyze variables associated with QOL. Physical and mental component scores (PCS and MCS) were higher among the stroke patients during the pandemic (44.20 ± 18.92 and 54.24 ± 19.08) than before the pandemic (37.98 ± 14.52 and 43.50 ± 20.94). Pandemic stress, demographic and clinical characteristics were negative variables associated with PCS and MCS. QPR was positively associated with PCS and MCS. The QOL of Chinese stroke patients was higher during than before the COVID-19 pandemic. Pandemic stress aggravated stroke patients’ QOL, while personal recovery could alleviate the detrimental effect of pandemic stress on QOL for stroke patients.

## Introduction

In 2018, stroke was the third most common cause of death for Chinese residents, with a death rate of 128/100,000 in urban residents and 160/100,000 in rural residents^1^. Strokes cause physical and psychological problems and can have a great impact on patients’ quality of life (QOL). Studies have been conducted to investigate the QOL of stroke patients and its related factors in different countries^2^. The poor QOL of patients with stroke is mainly related to personal and social factors and their recovery from the disease^3^. However, the QOL of stroke patients during the COVID-19 pandemic and the factors influencing this have not yet been studied, and this is an area in need of further exploration.

During the COVID-19 pandemic, the uncertainty and complexity of the situation, as well as a lack of knowledge about the causes and prevention of the disease, are likely to result in pandemic stress and may further affect patients’ quality of life^4^. Manifestations of pandemic stress can result in depression, phobia, and somatization^5^, cause neurological deficits, delay the process of recovery, and affect QOL.

Previous studies have reported that stroke patients with high activities of daily living (ADL) scores experienced poor QOL^6,7^. ADLs can have a major impact not only on patients’ physical health but also on their mental QOL^8^. Saunders et al.^9^ found that physical activity can promote the mental health of patients with stroke and increase their independence.

Personal recovery refers to a kind of positive psychological state in an individual in the process of recovering from mental illness^10^. Personal recovery has been reported as a positive resource for improving patients’ well-being over time. Psychological rehabilitation has an impact on patients’ QOL after a stroke. Research has shown that psychological interventions and rehabilitation training are effective for stroke rehabilitation and may further improve patients’ QOL^11^.

Most of the pandemic-related studies recently published in China have focused on medical staff or COVID-19-infected patients; however, there have been relatively few studies specifically concerning the physical and mental QOL of patients with chronic disease in the context of the pandemic, especially stroke patients. To our knowledge, few studies have yet been conducted to explore the factors related to the QOL of patients with stroke during the COVID-19 pandemic. This study therefore aims to compare the QOL of Chinese patients with stroke before and during the COVID-19 pandemic and explore associated variables, especially the effects of pandemic stress and personal recovery. The results may provide evidence to inform a theoretical basis for developing a strategy to improve the QOL of patients with stroke in the face of the COVID-19 pandemic.

## Results

### Demographic and clinical characteristics

Of a total of 479 patients, 344 patients (172 pairs before and during the pandemic) were identified in the statistical matching process as suitable for inclusion. The matched groups did not differ in their matching variables before and during the pandemic, including ADL and chronic disease. By comparing these data, we found that there were no significant differences in age, gender, marital status, educational level, or monthly income between the two periods. These demographic and clinical characteristics are shown in Table 1.

**Table 1:** Comparisons of PCS and MCS by demographic and clinical characteristics of patients with stroke (*n* = 344).

Variable	Before the pandemic (*n* = 172)						During the pandemic(*n* = 172)					Total(*n* = 344)			
	***n*(%)**	**PCS**		**MCS**		***n* (%)**	**PCS**		**MCS**		***n*(%)**	**PCS**		**MCS**	
		**M ± SD**	*p*	**M ± SD**	*p*		**M ± SD**	*p*	**M ± SD**	*p*		**M ± SD**	*p*	**M ± SD**	*p*
**Age (years)**															
≤ 60	87 (50.58)	41.66 ± 16.04**	0.001	48.78 ± 20.34**	0.001	50 (29.07)	47.09 ± 17.84	0.200	59.56 ± 19.45**	0.019	137 (39.83)	43.64 ± 16.86*	0.021	52.71 ± 20.62**	0.005
> 60	85 (49.42)	34.22 ± 11.72		38.10 ± 20.26		122 (70.93)	43.01 ± 19.29		52.06 ± 18.58		207 (60.14)	39.40 ± 17.12		46.33 ± 20.43	
**Gender**															
Male	114 (66.28)	38.42 ± 14.51	0.579	43.96 ± 21.81	0.689	109 (63.37)	43.45 ± 19.18	0.500	54.06 ± 19.49	0.872	223 (64.83)	40.88 ± 17.10	0.760	48.90 ± 21.28	0.975
Female	58 (33.73)	37.12 ± 14.61		42.60 ± 19.25		63 (36.63)	45.48 ± 18.54		54.55 ± 18.52		121 (35.14)	41.47 ± 17.22		48.83 ± 19.73	
**Marital status**															
Married	160 (93.02)	37.44 ± 14.08	0.070	42.80 ± 20.95	0.110	154 (89.52)	44.25 ± 19.29	0.912	54.56 ± 19.46	0.525	314 (91.28)	40.78 ± 17.16	0.275	48.57 ± 21.04	0.381
Other	12 (6.98)	45.29 ± 18.61		52.81 ± 19.26		18 (10.47)	43.73 ± 15.78		51.53 ± 15.81		30 (8.73)	44.35 ± 16.67		52.04 ± 16.96	
**Education level**															
High school or above	102 (59.30)	40.64 ± 14.49**	0.003	46.41 ± 21.81**	0.024	79 (45.93)	47.34 ± 19.67*	0.044	54.61 ± 18.13	0.815	181 (52.62)	43.57 ± 17.22**	0.005	49.99 ± 20.64	0.293
Junior school or under	70 (40.70)	34.11 ± 13.74		39.27 ± 18.96		93 (54.07)	41.52 ± 17.93		53.93 ± 19.96		163 (47.38)	38.34 ± 16.63		47.63 ± 20.79	
**Monthly income (RMB)**															
≤ 3000	58 (33.72)	33.61 ± 15.36	0.018	35.53 ± 17.05	0.001	72 (41.86)	44.58 ± 19.28	0.451	53.01 ± 17.44	0.331	130 (37.79)	39.69 ± 18.40	0.269	45.22 ± 19.29	0.029
3001–6000	76 (44.19)	40.19 ± 14.34**		46.58 ± 20.16**		90 (52.33)	44.71 ± 18.88		55.96 ± 20.75		166 (48.26)	42.64 ± 17.06		51.66 ± 21.22*	
> 6000	38 (22.09)	40.21 ± 14.52*		49.51 ± 23.40**		10 (5.81)	36.84 ± 16.64		47.64 ± 13.37		48 (13.95)	39.51 ± 13.10		49.12 ± 21.59*	
**Chronic diseases**															
> 3	118 (68.60)	38.99 ± 14.97	0.180	43.58 ± 21.16	0.940	118 (68.60)	42.35 ± 15.74	0.105	53.51 ± 19.30	0.456	236 (68.60)	40.67 ± 15.42	0.542	48.55 ± 20.81	0.669
≤ 2	54 (31.40)	35.79 ± 13.33		43.32 ± 20.34		54 (31.40)	48.24 ± 24.15		22.83 ± 18.69		108 (31.40)	42.01 ± 20.40		49.58 ± 20.58	
**ADL**															
Mild disability	50 (29.07)	51.48 ± 14.26**	0.001	53.12 ± 19.73**	0.001	68 (39.53)	57.45 ± 19.66**	0.001	56.73 ± 17.55	0.168	118 (34.30)	54.92 ± 17.76**	0.001	55.20 ± 18.50**	0.001
High disability	122 (70.93)	32.45 ± 10.46		39.56 ± 20.20		104 (60.47)	35.53 ± 12.28		52.61 ± 19.95		226 (65.70)	33.87 ± 11.41		45.57 ± 21.07	
**Types of stroke**															
Intracerebral hemorrhage	50 (29.07)	36.36 ± 15.24		42.99 ± 20.44		39 (22.67)	35.26 ± 16.74		48.40 ± 18.85		89 (25.87)	35.88 ± 15.83		45.37 ± 19.83	0.064
Cerebral infarction	122 (70.93)	38.65 ± 14.22	0.348	43.71 ± 21.22	0.840	133 (77.33)	46.81 ± 18.77**	0.001	55.95 ± 18.89**	0.029	255 (74.13)	42.91 ± 17.21**	0.001	50.10 ± 20.91	
**Hemiplegia**															
Y	163 (94.77)	37.18 ± 14.24	0.002	43.03 ± 20.88	0.208	117 (68.02)	39.19 ± 15.91	0.001	51.67 ± 17.77	0.009	280 (81.39)	38.02 ± 14.97	0.001	46.64 ± 20.06	0.001
N	9 (5.23)	52.51 ± 11.91**		52.07 ± 21.36		55 (31.68)	54.85 ± 20.47**		59.72 ± 20.76**		64 (18.60)	54.52 ± 19.44**		58.64 ± 20.84**	
**Aphasia**															
Y	44 (25.59)	33.23 ± 15.52	0.011	31.80 ± 17.58	0.001	47 (27.32)	36.78 ± 17.11	0.001	52.24 ± 16.88	0.401	91 (36.45)	35.06 ± 16.37	0.001	42.36 ± 19.97	0.001
N	128 (74.41)	39.62 ± 13.84**		47.52 ± 20.54**		125 (72.67)	46.98 ± 18.87**		54.99 ± 19.87		253 (73.55)	43.26 ± 16.90**		51.22 ± 20.51**	
**Dysphagia**															
Y	6 (3.49)	25.90 ± 5.54	0.038	22.42 ± 4.53	0.012	64 (37.21)	42.02 ± 18.65	0.246	57.49 ± 20.34	0.086	70 (20.35)	40.64 ± 18.45	0.805	54.49 ± 21.84**	0.011
N	166 (96.51)	38.42 ± 14.56**		44.26 ± 20.90**		108 (62.79)	45.49 ± 19.04		52.32 ± 18.13		274 (79.65)	41.20 ± 16.80		47.44 ± 20.21	

*p < 0.05, **p < 0.01.

The mean age of the participants was 61.57 ± 12.67 years, and 64.83% (*N* = 223) of the participants were male. Most participants were married (*N* = 314, 91.28%). The education level of 52.62% of the respondents was high school or higher, and that of 47.38% was junior high school or lower. In total, 37.79% of participants had monthly incomes of less than 3000 RMB, 48.26% had monthly incomes of 3001–6000 RMB, and 13.95% had monthly incomes of more than 6000 RMB. Most participants had three or more chronic diseases (*N* = 236, 68.60%). The mean ADL score of the participants was 24.10 ± 13.98. Approximately 29.07% of participants had intracerebral hemorrhage (*N* = 50) and 70.93% had cerebral infarction (*N* = 122). Most participants had hemiplegia (*N* = 163, 94.77%), while a few of them had aphasia (*N* = 44, 25.59%) or dysphagia (*N* = 6, 3.49%).

### Comparisons of stroke patients’ QOL and QPR scores

Comparisons of PCS and MCS based on demographic characteristics are shown in Table 1. Participants older than 60 years with a lower educational level, lower monthly incomes, higher disability, intracerebral hemorrhage, hemiplegia, aphasia or dysphagia were found to have lower PCS or MCS. There were no significant differences in PCS and MCS relating to gender, marital status, and chronic conditions.

**Figure 1:**
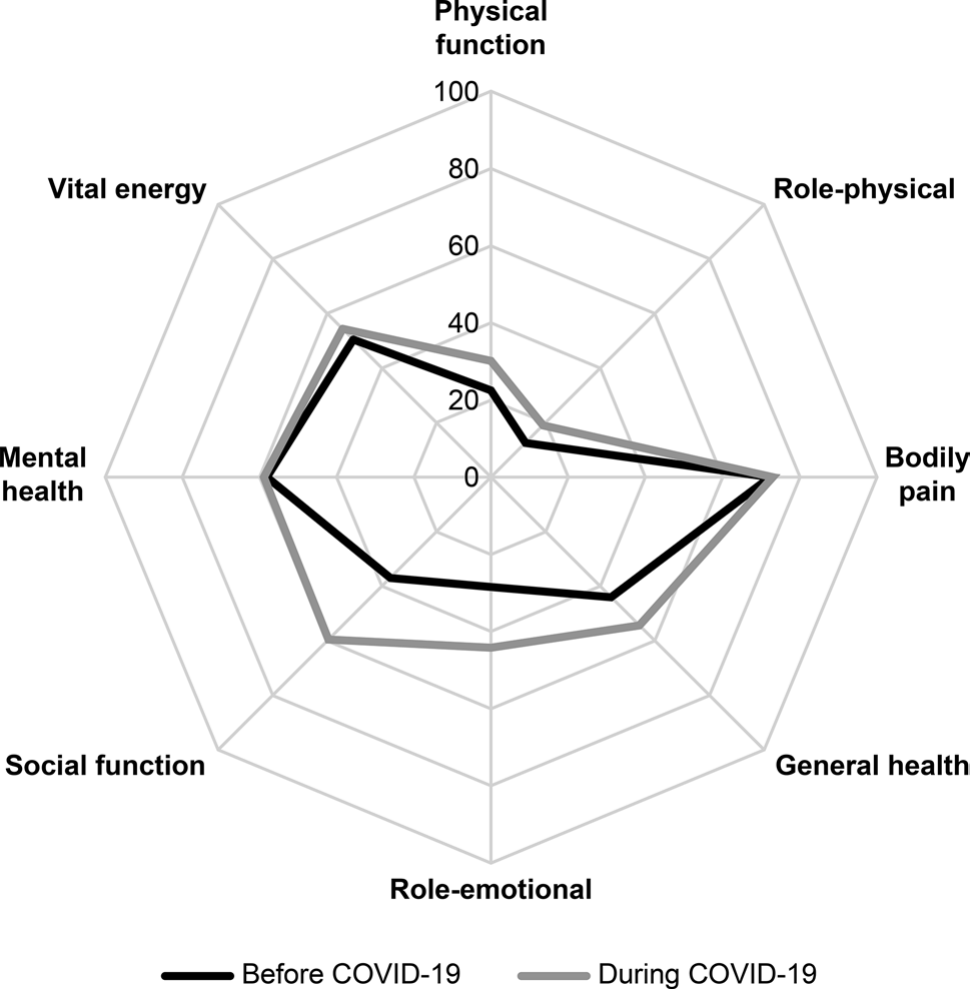
Comparison of stroke patients’ QOL before and during the COVID-19 pandemic. The overall PCS, MCS and the PF, RP, GH, RE, SF, VT sub-scores were significantly higher during the pandemic than those before the pandemic.

Comparisons of PCS and MCS before and during the COVID-19 pandemic are shown in Fig. 1. Before the pandemic, the mean PCS and MCS values among the patients with stroke were 37.98 ± 14.52 and 43.50 ± 20.94, respectively. During the pandemic, these scores were 44.20 ± 18.92 and 54.24 ± 19.08. The overall PCS and MCS and the PF, RP, GH, RE, SF, VT sub-scores were significantly higher during the pandemic than before the pandemic.

Comparisons of PCS and MCS considering thoughts about the pandemic are shown in Table 2. For each item, scores > 3 were used as grouping criteria for pandemic stress. During the pandemic, more than half of the participants felt that the impacts of the pandemic on their daily life were very low, low, or medium (*N* = 100, 58.14%), and less than half of them felt that the impacts were high or very high (N = 72, 41.86%). Most of the participants felt that the risk of infection was very low, low, or medium (*N* = 123, 71.51%), and few of them felt that the risk was high or very high (N = 49, 28.49%). Most of the participants felt that their worries about the pandemic were very low, low, or medium (*N* = 126, 73.26%), and few of them felt that their worries were high or very high (*N* = 46, 26.74%). The comparisons showed that participants with lower pandemic stress had a significantly higher PCS and/or MCS.

**Table 2:** Comparisons of PCS, MCS and QPR during the COVID-19 pandemic by thoughts about the pandemic (M ± SD, *n* = 172).

Variable	Felt that the “impacts of the pandemic on daily life were high or very high”			Felt that the “risks of contracting COVID-19 were high or very high”			Felt that “worries about the pandemic were high or very high”		
	**Y**	**N**	*p*	**Y**	**N**	*p*	**Y**	**N**	*p*
*n* **(%)**	72 (41.86)	100 (58.14)		49 (28.49)	123 (71.51)		46 (26.74)	126 (73.26)	
**PCS**	40.41 ± 15.91	49.45 ± 21.46**	0.003	38.07 ± 14.34	46.63 ± 19.99**	0.002	38.75 ± 12.17	46.18 ± 20.52**	0.004
PF	26.35 ± 17.62	35.35 ± 23.69	0.056	19.49 ± 12.30	34.35 ± 22.38**	0.001	24.13 ± 14.57	32.30 ± 22.26	0.080
RP	12.50 ± 8.98	28.13 ± 12.36**	0.008	12.76 ± 7.06	21.54 ± 18.71	0.094	13.59 ± 9.68	21.03 ± 17.87	0.181
BP	69.78 ± 26.24	77.47 ± 26.76	0.062	65.99 ± 24.58	75.79 ± 27.03**	0.029	65.22 ± 24.07	75.84 ± 27.07*	0.015
GH	53.03 ± 12.71	56.85 ± 12.54	0.052	54.06 ± 13.04	54.83 ± 12.67	0.714	52.07 ± 7.73	55.56 ± 14.05*	0.041
**MCS**	48.74 ± 16.91	61.88 ± 19.41**	0.001	50.62 ± 15.45	55.69 ± 20.23	0.079	47.84 ± 13.18	56.58 ± 20.38**	0.001
RE	33.33 ± 45.93	59.26 ± 47.87**	0.001	37.42 ± 26.96	46.88 ± 24.82	0.241	31.16 ± 9.68	48.94 ± 28.93*	0.027
SF	53.00 ± 21.12	58.67 ± 21.21**	0.001	53.74 ± 22.14	61.88 ± 22.27*	0.032	53.38 ± 17.47	61.82 ± 23.71*	0.013
MH	57.08 ± 11.95	61.33 ± 12.12**	0.023	58.86 ± 10.83	58.86 ± 12.71	0.998	55.83 ± 9.76	59.97 ± 12.79*	0.048
VT	51.55 ± 11.58	58.26 ± 13.97**	0.001	52.45 ± 10.16	55.12 ± 13.97	0.167	50.98 ± 6.72	55.60 ± 14.50**	0.005
**QPR**	57.61 ± 12.70	61.03 ± 10.60	0.057	60.69 ± 10.03	58.38 ± 12.61	0.209	55.87 ± 12.84	60.20 ± 11.45*	0.035

*p** < **0.05, **p** < **0.01.

The mean QPR score of the participants was 56.74 ± 13.26 (Table 2). Patients had higher QPR scores during the pandemic (59.04 ± 11.95) than before the pandemic (54.45 ± 14.11). During the pandemic, those who felt that their worries about the pandemic were high or very high had lower QPR scores.

### Associated factors of QOL

In the Spearman correlation analysis (Table 3), the QPR was positively associated with PCS (*p* < 0.001), MCS (*p* < 0.001), and the eight concepts of QOL (*p* < 0.001).

**Table 3:** Variables of the study during the pandemic (*n* = 172).

	1	2	3	4	5	6	7	8	9	10
**1.PCS**	1									
2.PF	0.799**	1								
3.RP	0.786**	0.493**	1							
4.BP	0.619**	0.318**	0.191*	1						
5.GH	0.512**	0.293**	0.265**	0.283**	1					
**6.MCS**	0.609**	0.288**	0.471**	0.525**	0.499**	1				
7.RE	0.530**	0.213**	0.503**	0.440**	0.294**	0.901**	1			
8.SF	0.465**	0.272**	0.243**	0.461**	0.461**	0.733**	0.446**	1		
9.MH	0.358**	0.164*	0.221**	0.321**	0.440**	0.674**	0.485**	0.415**	1	
10.VT	0.464**	0. 272**	0.265**	0.346**	0.633**	0.624**	0.345**	0.526**	0.504**	1
**11.QPR**	0.309**	0.184*	0.195*	0.207**	0.411**	0.407**	0.338**	0.263**	0.364**	0.338**

*p** < **0.05, **p** < **0.01.

**Figure 2:**
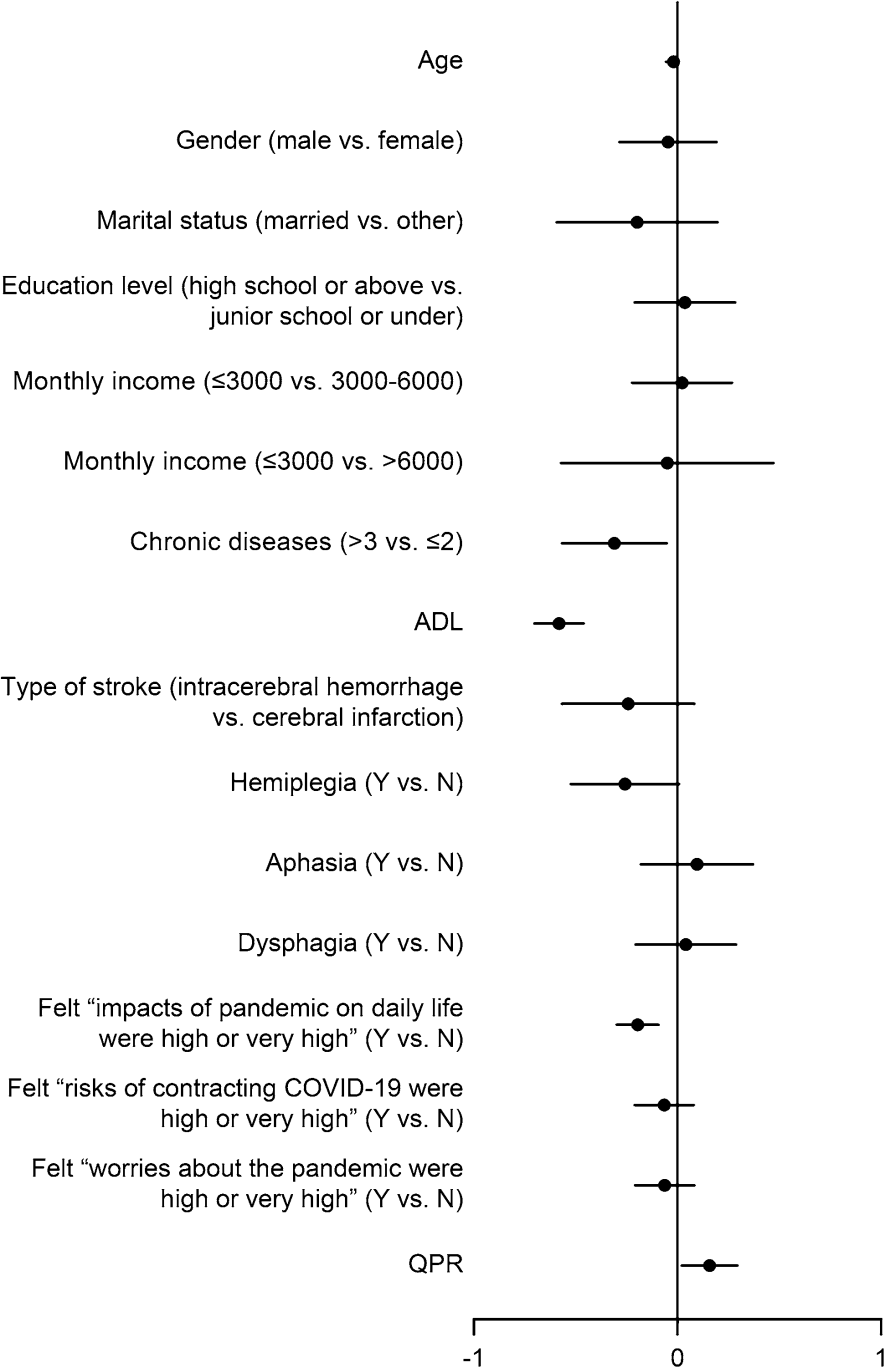
Forest plot of PCS.

For the PCS, we carried out a three-step HMR (Table 4). In the first step, the demographic and clinical characteristics were in the model. Adjusted R^2^ was 0.474, which accounted for 83.8% (0.474/0.568) of the model. When pandemic stress was added in the model, adjusted R^2^ was 0.557. The change of R^2^ was 0.083, which meant that 14.6% (0.083/0.568) of PCS could be caused by pandemic stress in the model. In the third step of HMR, QPR was entered in the model. Adjusted R^2^ was 0.568 and ΔR^2^ was 0.011, which meant that 1.9% (0.011/0.568) of PCS could be explained by the QPR in this model. Among all variables measured in this study, PCS was significantly associated with, in the sequence of *β* value, ADL (*β* =  − 0.587, *p* = 0.001), felt that the “impacts of the pandemic on daily life were high or very high” (*β* =  − 0.231, *p* = 0.001), chronic disease (*β* =  − 0.131, *p* = 0.018), QPR (*β* = 0.129, *p* = 0.024) and age (*β* =  − 0.113, *p* = 0.045) (Fig. 2).

**Table 4:** Hierarchical multiple regression of PCS and MCS during the pandemic (*n* = 172).

**Variable**		**PCS**			**MCS**		
		Step 1(*β)*	Step 2(*β)*	Step 3(*β)*	Step 1(*β)*	Step 2(*β)*	Step 3(*β)*
**Demographic and clinical characteristics**							
Age		− 0.118	− 0.110	− 0.113*	− 0.174*	− 0.137	− 0.141*
Gender	(Male vs. Female)	0.016	− 0.020	− 0.020	0.001	− 0.064	− 0.065
Marital status	(Married vs. Other)	− 0.012	− 0.048	− 0.055	− 0.035	− 0.094	− 0.110
Education level	(High school or above vs. Junior school or under)	− 0.001	0.033	0.016	− 0.068	− 0.024	− 0.059
Monthly income	(≤ 3000 vs. 3000–6000)	0.024	0.009	0.010	0.090	0.041	0.044
	(≤ 3000 vs. > 6000)	− 0.007	0.012	− 0.011	− 0.003	0.014	− 0.036
Chronic diseases	(> 3 vs. ≤ 2)	− 0.126*	− 0.135*	− 0.131*	− 0.044	− 0.048	− 0.039
ADL		− 0.589**	− 0.606**	− 0.587**	− 0.123	− 0.163	− 0.122
Types of stroke	(Intracerebral hemorrhage vs. Cerebral infarction)	− 0.129	− 0.109	− 0.092	− 0.190*	− 0.195*	− 0.159*
Hemiplegia	(Y vs. N)	− 0.136*	− 0.123*	− 0.109	− 0.131	− 0.111	− 0.080
Aphasia	(Y vs. N)	0.021	0.039	0.039	0.028	0.052	0.051
Dysphagia	(Y vs. N)	0.036	0.020	0.018	− 0.158*	− 0.143*	− 0.138*
**Pandemic stress**							
Felt that the “impacts of the pandemic on daily life were high or very high”	(Y vs. N)		− 0.255**	− 0.231**		− 0.404**	− 0.353**
Felt that the “risks of contracting the COVID-19 were high or very high”	(Y vs. N)		− 0.084	− 0.055		− 0.271**	− 0.208**
Felt that “worries about the pandemic were high or very high”	(Y vs. N)		0.006	0.044		− 0.208**	− 0.124
**QPR**				0.129*			0.284**
*Ɛ*		1.817	1.590	1.403	2.125	2.833	2.489
Adjusted R^2^		0.474**	0.557**	0.568**	0.066	0.303**	0.369**
△R^2^		0.474	0.083	0.011	0.066	0.237	0.066

Adjusted R^2^: A changed variation of R^2^ that has been changed for the number of variables. The adjusted R^2^ rises when the model improves with new blocks of variables; ΔR^2^:An index for measuring the proportion of the variability of the single variable or block that is explained by the models; *β*: the partial regression coefficient of the variables, which indicates the amount by which the dependent variable increases or decreases for each change of a specified independent variable by a unit; *Ɛ*: Constant; *p < 0.05, **p < 0.01.

The study variables of PCS are shown in the forest plot. Age, chronic disease, ADL, felt that the “impacts of pandemic on daily life were high or very high” were negatively associated with PCS, while QPR was positively associated with PCS.

**Figure 3:**
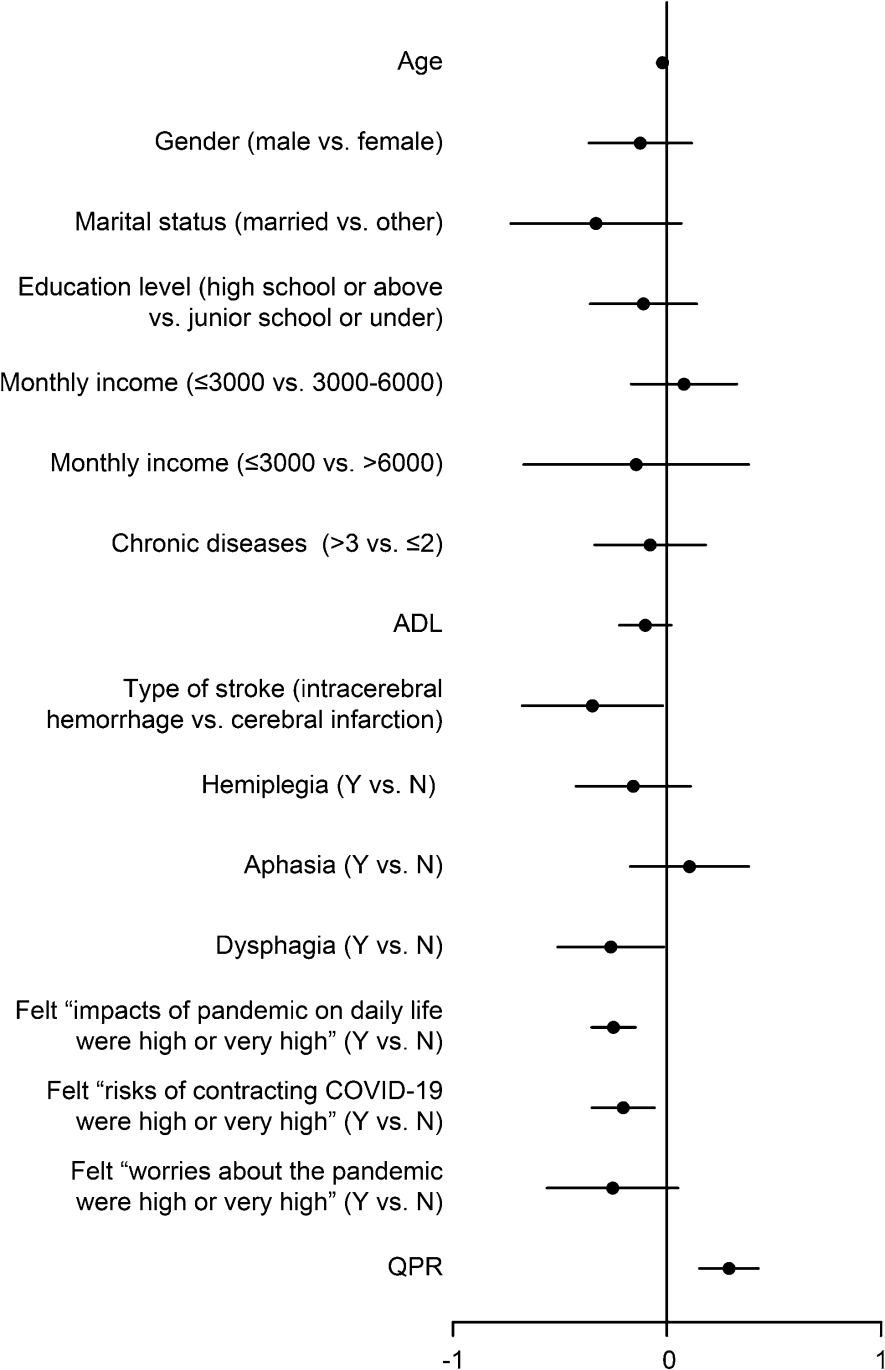
Forest plot of MCS.

For the three-step MCS regression model (Table 4), the demographic and clinical characteristics was added in the model for the first step. Adjusted R^2^ was 0.066, which meant that 17.9% of MCS could be explained by the variables of demographic and clinical characteristics in the model. In the second step of HMR, pandemic stress was added in the model. Adjusted R^2^ was 0.303, The change of R^2^ was 0.237, which meant that 64.2% of MCS could be explained by the new added variables of pandemic stress in the model. In the third step of HMR, QPR was new added in the model. Adjusted R^2^ was 0.369 and ΔR^2^ was 0.066, which meant that 17.9% of MCS could be explained by QPR in the model. Among all variables measured in this study, MCS was significantly associated with, in the sequence of *β* value, felt that the “impacts of the pandemic on daily life were high or very high” (*β* =  − 0.353, *p* = 0.001), QPR (*β* = 0.284, *p* = 0.001), felt that the “risks of contracting the COVID-19 were high or very high” (*β* =  − 0.208, *p* = 0.006), stroke type (*β* =  − 0.159, *p* = 0.036), age (*β* =  − 0.141, *p* = 0.037) and dysphagia (*β* =  − 0.138, *p* = 0.037) (Fig. 3).

The study variables of MCS are shown in the forest plot. Age, stroke type, dysphagia, felt that the “impacts of pandemic on daily life were high or very high”, felt that the “risks of contracting the COVID-19 were high or very high” were negatively associated with PCS, while QPR was positively associated with PCS.

**Figure 4:**
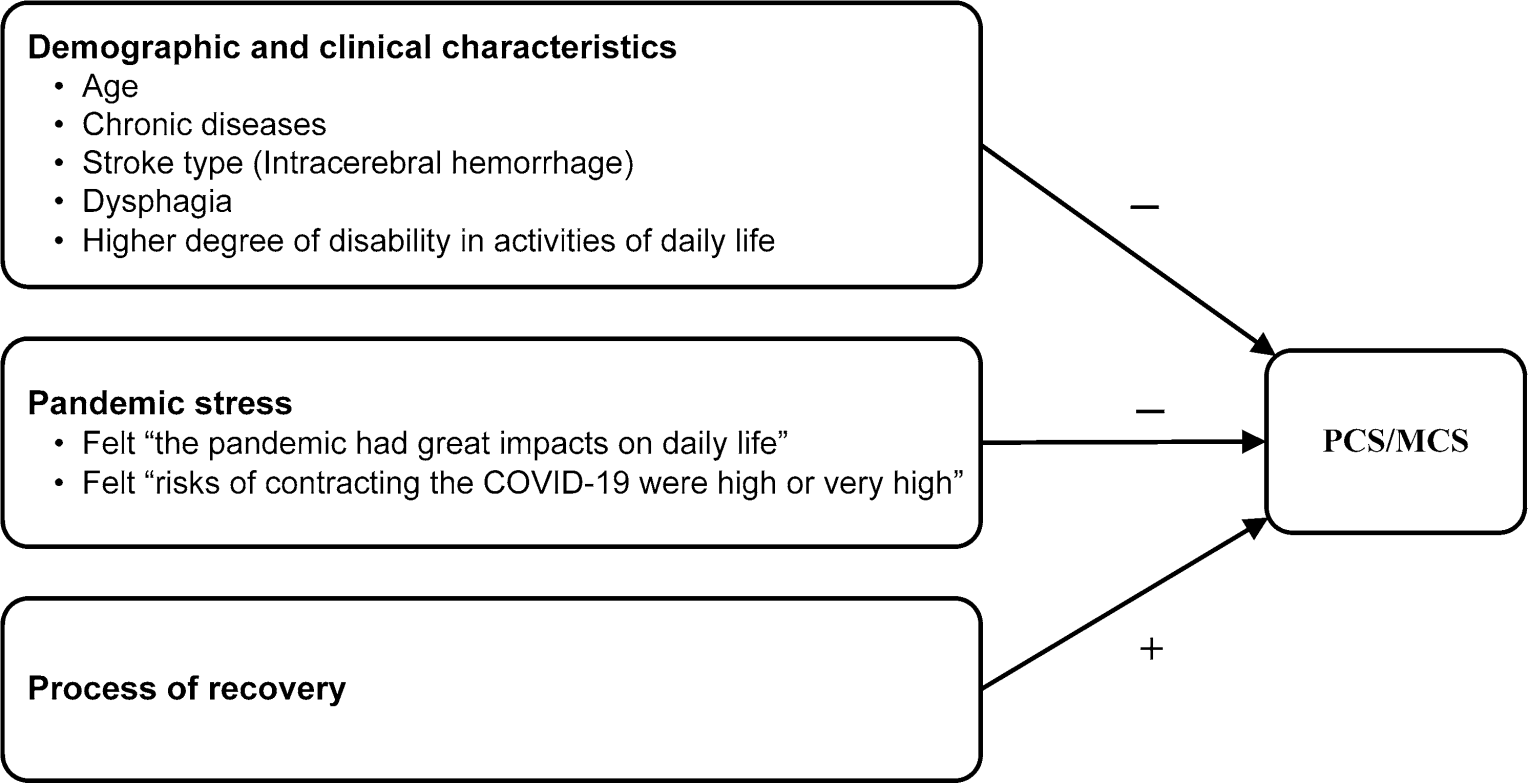
Variables associated with stroke patients’ QOL during the COVID-19 pandemic.

In summary, stroke patients’ basic characteristics (age, chronic disease, stoke type, dysphagia, ADL), pandemic stress and QPR were variables associated with PCS and MCS during the pandemic (Table 4 and Fig. 4).

Stroke patients’ basic characteristics (age, chronic disease, stoke type, dysphagia, ADL) and pandemic stress were negatively associated with PCS and MCS, while QPR were positively associated with PCS and MCS during the pandemic.

## Discussion

We conducted a survey on the QOL of patients with stroke in the second half of 2019, but it was interrupted by the outbreak of the COVID-19 pandemic. Our study therefore had to be postponed until April 2020, and the study was by chance the first to compare the QOL of patients with stroke before and during the COVID-19 outbreak. Surprisingly, we found that during the pandemic, the PCS and MCS values of the patients with stroke in China were higher than those before the pandemic. One reason for this might be improvements in patients’ personal recovery experience and stroke treatment. As an indicator of personal recovery on mental health status, QPR was a variable associated with stroke patients’ QOL, explaining 1.9% of the variance in PCS and 17.9% of the variance in MCS. Comparisons showed that QPR scores were higher during the pandemic than before the pandemic. This might be a result of the relatively serene and spacious medical environment that was necessary for disease prevention and control. During the pandemic, medical institutions were more isolated and safer for noninfectious patients because of a variety of prevention measures, such as strengthening visit management and reducing the number of accompanying staff. According to statistics from the National Health Commission, during the pandemic, the utilization rate of hospital beds was only 62.2% in the first quarter of 2020, representing a year-on-year decrease of 22.8%^12^. Noninfectious patients were also arranged in separate rooms as much as possible, and strict disinfection measures were taken; thus, the patients could receive appropriate care and treatment, which resulted in the enhancement of recovery ability and perceived QOL. A relatively sparse medical hardware environment, a sense of a lower chance of infection inside than outside the hospital, and more intensive visits by medical staff might be reasons for the observed increase in QPR scores and may have positively influenced the QOL of patients with stroke.

This study was the first to evaluate the QOL of patients with stroke during the COVID-19 pandemic and test the related risk factors. We found that pandemic stress, personal recovery, demographic and clinical characteristics, such as age, chronic disease, stroke type, dysphagia and ADL were associated with stroke patients’ QOL. For pandemic stress, 14.6% of the variance in PCS and 64.2% of the variance in MCS was explained; participants with lower pandemic stress had significantly higher PCS and MCS values. We also found that Chinese patients with stroke experienced a relatively low level of stress and higher QOL during the pandemic. This may be due to the rapid and comprehensive public health emergency interventions of the Chinese government, as well as the efforts to reduce panic and misunderstanding among the public. Another study on home-quarantined Chinese university students provided similar insights into posttraumatic stress disorder (PTSD) during the COVID-19 pandemic in China, revealing a much lower rate of PTSD than that in other countries during the SARS pandemic^13^. Demographic and clinical characteristics, such as age, chronic disease, stroke type, dysphagia and ADL explained 83.8% of the variance in PCS and 17.9% of the variance in MCS during the pandemic in the model.

Although all populations are susceptible to COVID-19 infection, particular attention should be paid to vulnerable groups, such as elderly people with chronic disease, especially those with stroke^14^. During the pandemic, patients with stroke experienced a relatively impaired QOL compared to the general Chinese population^15^. We make the following suggestions to improve the QOL of these patients. First, it is crucial to take measures to prevent and reduce pandemic stress. The provision of guidance about the pandemic situation and the dissemination of public health information should be strengthened with urgency. Second, appropriate functional training and psychological counseling should be provided to combat pandemic-related stress and improve QOL for stroke patients. The enhancement of personal recovery could be a new intervention strategy to improve the QOL of patients with stroke. Third, it is vital to enhance the quality of care for stroke patients. A better healthcare service environment should be built to help reduce the negative emotions associated with the pandemic and eventually increase the patients’ well-being. More attention should also be paid to older patients with lower educational levels or low income.

The study has certain limitations. There was the possibility of the bias that different hospitals were visited before and during the pandemic. This was because during the pandemic, movement between provinces and cities to conduct the survey was limited by pandemic control strategies, and we could only visit local hospitals. Furthermore, to decrease the time required to conduct the survey, we increased the number of hospitals surveyed. Considering the above factors, we took three measures to mitigate any potential bias: (i) we carefully compared and selected the hospitals surveyed during the pandemic, choosing only hospitals capable of stroke care in the local “stroke emergency map” that were highly homogeneous with respect to those surveyed before the pandemic; (ii) we strictly controlled the inclusion and exclusion criteria for the patients so that the study subjects would be representative; and (iii) we used a matched-pair study, matching participants before and during the pandemic to ensure homogeneity. Besides, we did not use the NIH Stroke Scale (NIHSS) to measure stroke-related neurologic deficit, and also did not collect abundant clinical details about stroke, therapy, artery involved, laterality etc. For the next study, we will pay more attention to collect clinical features of the stroke patients.

## Methods

### Study design and sample

We conducted a multicenter survey in Liaoning Province and Chongqing municipality of China between August and December 2019, and two territories of general hospitals were visited (Chongqing General Hospital and Shengjing Hospital of China Medical University). However, the sudden nationwide outbreak of COVID-19 interrupted this survey^16^. We continued the survey in April in the local province of Liaoning until the pandemic slowed. We chose four territorial general hospitals that were similar to the previous research hospitals (the First, Fourth and Fifth Hospitals of Shenyang, the Second Affiliated Hospital of Shenyang Medical College). During the outbreak, these hospitals were not designated for the treatment of infected patients. These six institutions are all designated hospitals in the “stroke emergency map” of local cities^17,18^, and their clinical level of neurology and neurosurgery has been certified by the local health administrative department.

Before the pandemic, 350 smartphone questionnaires were distributed to stroke inpatients in the hospital, and 272 participants answered the smartphone questionnaire with the help of the surveyors. During the pandemic, matched participants with a similar severity of stroke (ADL score) and a similar chronic condition were then surveyed. In this phase, 300 smartphone questionnaires were distributed, and 207 participants completed the survey. The overall response rate was 73.69%. The inclusion criteria were patients (i) with a diagnosis of ischemic or hemorrhagic stroke made by a clinician and/or supported by brain imaging; (ii) with a recent stroke occurring within four weeks and in stable condition of the recovery stage; and (iii) able to understand spoken or written language and able to answer using language or gestures. The exclusion criteria were as follows: (i) severe condition (massive cerebral infarction or hemorrhage, serious myocardial infarction or heart surgery, etc.); (ii) pre-existing neurological disorders, such as Parkinson’s disease, multiple sclerosis, or severe dementia; (iii) severe uncontrolled psychiatric illness or acute infectious diseases; or (iv) a history of sustained alcoholism or drug abuse in the last six months.

All the patients involved participated anonymously and signed an online consent form about the contents of the questionnaire. The participants completed the online questionnaires using mobile phones via the Chinese Wenjuanxing platform. The research was conducted in accordance with the Helsinki Declaration as revised in 1989, and the study protocol was approved by the Ethics Committee of China Medical University. All subjects gave their informed consent online for inclusion before participating in the study.

### Measurements

#### Demographic and clinical characteristics

Demographic and clinical characteristics regarding age, gender, marital status, education, monthly income, chronic diseases, ADL, stroke type, hemiplegia, aphasia and dysphagia were included. “Marital status” was categorized as “married” or “other” (including single, divorced, etc.). “Education” was categorized as either “high school or above” or “junior school or under.” “Monthly income” (RMB) was categorized as “ ≤ 3000,” “3001–6000,” and “ > 6000.” “Chronic diseases” was categorized as either “ > 3” (had three or more chronic diseases) or “ ≤ 2” (had less than 2 chronic diseases). The ADL score was used to categorize patients into “mild disability” (ADL score ≤ 21) or “high disability” (ADL score > 21). It is a 14-item questionnaire designed to measure the degree of disability in completing certain activities, such as walking, cooking, and dressing^19^. It is a four-point Likert scale, with total scores range from 0 to 42, and higher scores indicate a higher level of disability^8^. The Cronbach’s alpha for the ADL score was 0.965 in this study. Stroke type was categorized as “cerebral infarction” or “intracerebral hemorrhage”. Hemiplegia, aphasia and dysphagia were categorized as either “Y” (had the manifestation) or “N” (did not have the manifestation).

#### Health-related QOL

The QOL of patients with stroke was measured using the 36-item Short-Form Health Survey (SF-36). This is a generic health questionnaire developed in the Medical Outcomes Study^20^. The Mandarin version of the SF-36 was translated by experts at Zhejiang University in China^21,22^. It consists of two components and eight concepts. The physical component score (PCS) contains four concepts: physical functioning (PF), role limitations due to physical problems (RP), bodily pain (BP), and general health (GH); the mental component score (MCS) also contains four dimensions: vitality (VT), social functioning (SF), role limitations due to emotional problems (RE), and general mental health (MH)^23^. The PCS and MCS values range from 0 to 100, and higher scores indicate better QOL. The Cronbach’s alpha values in this study were 0.758 for the complete SF-36 and 0.612 and 0.611 for the PCS and MCS, respectively.

#### Pandemic stress

Pandemic stress was measured using a questionnaire specifically designed for this study, which consisted of three items: (i) how patients felt about the impacts of the COVID-19 pandemic on daily life; (ii) how patients felt about the risks of contracting COVID-19; and (iii) how worried patients felt about the pandemic. For each item, the scale ranged from 1 (very low) to 5 (very high). The Cronbach’s alpha for the questionnaire was 0.678.

#### Process of recovery

Mental recovery was measured using the Questionnaire about the Process of Recovery (QPR)^10^ and was translated into Chinese^24^. It consists of 22 items and two factors: an intrapersonal factor (17 items) and an interpersonal factor (5 items)^25^. It is a five-point Likert scale, and total scores range from 0 to 88, with higher scores indicating better recovery. It was used to measure the recovery of psychotic patients in process and outcome evaluation studies. However, it can also be applied to the assessment of recovery across a diverse range of diseases^26^. The Cronbach’s alpha for the QPR in this study was 0.965.

### Matched-pair analysis

To investigate differences in the QOL of patients with stroke before and during the pandemic, a matched-pair analysis was conducted^27^. We performed the sensitivity analysis by applying propensity score matching. Two potentially confounding variables, ADL score and chronic disease status, were used for exact matching. During the matching (one-to-one without replacement) process, the maximum allowed difference in ADL score was defined as less than 1 SD of ADL scores, and the patients’ chronic disease status had to be the same as that of ADL.

### Statistical analysis

Differences in the QOL measured by the PCS and MCS parts of the SF-36 were compared considering the patients’ demographic and clinical characteristics, before and during the COVID-19 pandemic, and patients’ stress about the pandemic. An independent samples *t*-test, one-way analysis of variance, and cross-tabulations were used to compare the differences. Spearman correlation was performed among continuous variables. HMR was carried out to analyze variables associated with PCS and MCS during the pandemic. A *p* value < 0.05 (two-tailed) was considered significant. Data were analyzed using SPSS v13.0. All study variables were standardized before analysis.

## Data Availability

The raw data supporting the conclusions of this article will be made available by the authors, without undue reservation, to any qualified researcher.

## References

[1] China NHC. *China health statistics yearbook 2019* (China Union Medical University Press, 2020).

[2] Labberton AS, Augestad LA, Thommessen B, Barra M (2020). The association of stroke severity with health-related quality of life in survivors of acute cerebrovascular disease and their informal caregivers during the first year post stroke: a survey study. Qual. Life Res..

[3] Pedersen SG (2020). Stroke-specific quality of life one-year post-stroke in two Scandinavian country-regions with different organisation of rehabilitation services: a prospective study. Disabil. Rehabil..

[4] Vindegaard N, Benros ME (2020). COVID-19 pandemic and mental health consequences: systematic review of the current evidence. Brain Behav. Immun..

[5] Liu X (2020). Psychological status and behavior changes of the public during the COVID-19 epidemic in China. Infect. Dis. Poverty.

[6] Li W (2019). Anxiety in patients with acute ischemic stroke: risk factors and effects on functional status. Front. Psychiatry.

[7] Mustafaoglu R, Erhan B, Yeldan I, Gunduz B, Tarakci E (2020). Does robot-assisted gait training improve mobility, activities of daily living and quality of life in stroke? A single-blinded, randomized controlled trial. Acta Neurol. Belg..

[8] Li H (2020). Prevalence of somatic-mental multimorbidity and its prospective association with disability among older adults in China. Aging (Albany NY).

[9] Saunders DH, Greig CA, Mead GE (2014). Physical activity and exercise after stroke: review of multiple meaningful benefits. Stroke.

[10] Neil ST (2009). The questionnaire about the process of recovery (QPR): a measurement tool developed in collaboration with service users. Psychosis.

[11] Stalder-Lüthy F (2013). Effect of psychological interventions on depressive symptoms in long-term rehabilitation after an acquired brain injury: a systematic review and meta-analysis. Arch. Phys. Med. Rehabil..

[12] China NHC. National medical services from January to March 2020. http://www.nhc.gov.cn/mohwsbwstjxxzx /s2906/new_list_2.shtml (2020).

[13] Tang W (2020). Prevalence and correlates of PTSD and depressive symptoms one month after the outbreak of the COVID-19 epidemic in a sample of home-quarantined Chinese university students. J. Affect. Disord..

[14] Wang H (2020). Potential mechanisms of hemorrhagic stroke in elderly COVID-19 patients. Aging (Albany NY).

[15] Zhu YB, Wang Q, Chen KF (2009). Predictors of health-related quality of life in the general population. Chin. J. Behav. Med. Brain Sci..

[16] China State Council. National emergency plan for public health emergencies. http://www.gov.cn/ (2006).

[17] Shenyang Daily. There are 32 hospitals in “Shenyang stroke emergency map”. http://www.ln.xinhuanet.com/2019-06/09/c_1124598624.htm (2019).

[18] Chongqing Stroke Society. The official version of “Chongqing stroke emergency map” is launched. http://www.cq.xinhuanet.com/2019-07/10/c_1124729782.htm (2019).

[19] Lawton MP, Brody EM (1969). Assessment of older people: self-maintaining and instrumental activities of daily living. Gerontologist.

[20] Ware JE, Sherbourne CD (1992). The MOS 36-item short-form health survey (SF-36). I. Conceptual framework and item selection. Med. Care.

[21] Wang R (2008). Health related quality of life measured by SF-36: a population-based study in Shanghai, China. BMC Public Health.

[22] Li L, Wang HM, Shen Y (2003). Chinese SF-36 health survey: translation, cultural adaptation, validation, and normalisation. J. Epidemiol. Community Health.

[23] Ware, J. E. & Sherbourne, C. D. *Measuring functioning and well-being: the medical outcomes study approach* (Duke University Press, 1992).

[24] Chien WT, Chan ZC (2013). Chinese translation and validation of the questionnaire on the process of recovery in schizophrenia and other psychotic disorders. Res. Nurs. Health.

[25] Law H, Neil ST, Dunn G, Morrison AP (2014). Psychometric properties of the questionnaire about the process of recovery (QPR). Schizophr. Res..

[26] Shanks V (2013). Measures of personal recovery: a systematic review. Psychiatr. Serv..

[27] Austin PC (2011). Optimal caliper widths for propensity-score matching when estimating differences in means and differences in proportions in observational studies. Pharm. Stat..

